# Myelodysplastic Syndromes‐Transformed Acute Myeloid Leukemia With Concurrent DEK::CAN Fusion Gene Positivity and WT1 Overexpression: A Case Report and Literature Review

**DOI:** 10.1002/cnr2.70714

**Published:** 2026-10-09

**Authors:** Wanxiu Mao, Tengmin Zhou, Xi Xu, Sheng Luo, Songfu Jiang, Zhijie Yu

**Affiliations:** ^1^ Zhejiang Chinese Medical University Hangzhou China; ^2^ Department of Hematology, Wenzhou Key Laboratory of Hematology Research The First Affiliated Hospital of Wenzhou Medical University Wenzhou China; ^3^ Department of Rehabilitation Medicine The First Affiliated Hospital of Wenzhou Medical University Wenzhou China

**Keywords:** case report, DEK::CAN fusion gene, MDS‐transformed acute myeloid leukemia, myelodysplastic syndromes, WT1 overexpression

## Abstract

**Background:**

Myelodysplastic syndromes (MDS) are a group of clonal hematopoietic stem cell disorders with a risk of progression to acute myeloid leukemia (AML). MDS‐transformed AML (MDS‐AML) has a poor prognosis. The concurrent presence of DEK::CAN fusion gene positivity and WT1 overexpression is extremely rare and likely denotes a highly aggressive disease.

**Case:**

A 57‐year‐old Han Chinese female with a 5‐year history of MDS (DEK::CAN status at initial diagnosis unknown) presented with fatigue and pancytopenia. Bone marrow blasts accounted for 81% (AML‐M5), and karyotype showed t(6;9)(p23;q34). Real‐time quantitative PCR (qPCR) revealed a DEK::CAN/ABL ratio of 188.21% and a WT1/ABL ratio of 52.90%; next‐generation sequencing identified a U2AF1 mutation (p.Arg156His). The patient received IA regimen induction followed by intermediate‐dose cytarabine consolidation. Complete remission (CR) was achieved after induction; after the first consolidation cycle, a transient CR with incomplete hematologic recovery (CRi) occurred, and CR was re‐established at the final evaluation. Severe infections occurred repeatedly during treatment. Although the patient achieved CR after induction chemotherapy, recurrent infections and cumulative chemotherapy‐related cytopenias prevented timely allogeneic hematopoietic stem cell transplantation (allo‐HSCT). She died approximately 1 month after the last chemotherapy cycle; the cause was clinically suspected to be fatal hemorrhage, though autopsy was not performed.

**Conclusion:**

MDS‐AML with concurrent DEK::CAN and WT1 mRNA overexpression is a highly aggressive entity. Although chemotherapy can induce remission, treatment‐related complications often preclude transplantation. Early molecular screening is recommended, and once any form of remission (CR or CRi) is achieved, prompt bridging to allo‐HSCT should be pursued. Targeted therapy should also be explored.

## 1 Introduction

Myelodysplastic syndromes (MDS) are a group of highly heterogeneous clonal hematopoietic stem cell disorders characterized by ineffective hematopoiesis and peripheral cytopenia, with a risk of progression to acute myeloid leukemia (AML) [1]. MDS‐transformed AML (MDS‐AML) accounts for approximately 15%–28% of all AML cases [2]; this category exhibits chemotherapy resistance and an extremely poor prognosis.

The DEK::CAN fusion gene arises from the balanced chromosomal translocation t(6;9)(p23;q34) and occurs in approximately 1%–2% of all AML patients [3]. This fusion gene drives leukemogenesis through dual mechanisms: on one hand, it aberrantly impairs nucleoporin function, disrupting nucleocytoplasmic transport and signal transduction [4]; on the other hand, it constitutively activates the mTOR signaling pathway, promoting malignant cell proliferation and inhibiting apoptosis [5]. The WT1 gene was initially identified as a tumor suppressor but often exerts oncogenic functions in hematologic malignancies; its overexpression has been detected in various myeloid neoplasms including MDS and AML [6, 7]. WT1 overexpression disrupts the balance between apoptosis and proliferation by inhibiting pro‐apoptotic genes (e.g., BAX) and aberrantly activating anti‐apoptotic pathways (e.g., PI3K/AKT), directly leading to malignant clone expansion and chemotherapy resistance [8, 9] Notably, the concurrent presence of DEK::CAN fusion gene positivity and WT1 overexpression in MDS‐AML is extremely rare, and the two abnormalities may synergistically promote disease progression through multiple signaling pathways and transcriptional regulatory networks.

Here we report in detail a case of MDS‐AML with DEK::CAN positivity and WT1 mRNA overexpression and perform a systematic literature review of published DEK::CAN‐positive MDS/AML cases [3, 10]. The aim is to improve clinical recognition of this rare disease entity, emphasize the importance of multiparameter molecular testing for accurate diagnosis and risk stratification, and explore optimal treatment strategies.

## 2 Case Report

### 2.1 Medical History and Initial Presentation

A 57‐year‐old Han Chinese female with a 5‐year history of MDS was found to have cytopenia 5 months before admission but did not seek medical intervention. She was admitted in August 2021 with a 3‐day history of fatigue and dizziness. Five years earlier, she had been diagnosed with “myelodysplastic syndrome” at an outside hospital and discharged after symptomatic treatment (details unknown). However, bone marrow samples or molecular test results from the initial MDS diagnosis could not be obtained for retrospective analysis; therefore, it is impossible to determine whether the DEK::CAN fusion gene was already present at the MDS stage or acquired upon transformation to AML. The patient denied any history of other malignancies, prior chemotherapy, or radiotherapy. Physical examination on admission showed only pale skin and mucous membranes, without hepatosplenomegaly or superficial lymphadenopathy.

### 2.2 Diagnostic Workup

1. Lab tests: Pancytopenia, WBC 0.68 × 10^9^/L, Hb 35 g/L, and PLT 23 × 10^9^/L.
2. Bone marrow morphology (Figure 1): The bone marrow aspirate smear showed marked hypercellularity with 81% blasts exhibiting monocytoid morphology: large cell bodies, abundant grayish‐blue cytoplasm, occasional cytoplasmic protrusions, a few fine granules, and no Auer rods; nuclei were regular or irregular with fine chromatin and large, distinct nucleoli (mostly single). The granulocytic and erythroid lineages were markedly suppressed (7.5% and 3%, respectively), and dysplastic changes were observed (occasional hypogranulation and megaloblastoid changes in the granulocytic lineage, and occasional megaloblastoid changes in the erythroid lineage). Megakaryocyte count was approximately 45 per whole smear, with acceptable morphology. Cytochemical staining: nonspecific esterase (NSE) 100% positive, strongly inhibited by sodium fluoride; peroxidase (POX) 50% positive. These morphological and cytochemical features were consistent with acute monocytic leukemia (AML‐M5). Compared with published DEK::CAN‐positive myeloid neoplasms, the present case showed the M5 subtype with multilineage dysplasia, which is consistent with common features in the literature [3]. Basophilia was once described as a feature of t(6;9), but a recent large‐scale study reported that only about 7% of cases exhibit basophilia [3]; the present case showed neither basophilia nor Auer rods, which also lies within the reported spectrum of variation.

**FIGURE 1:**
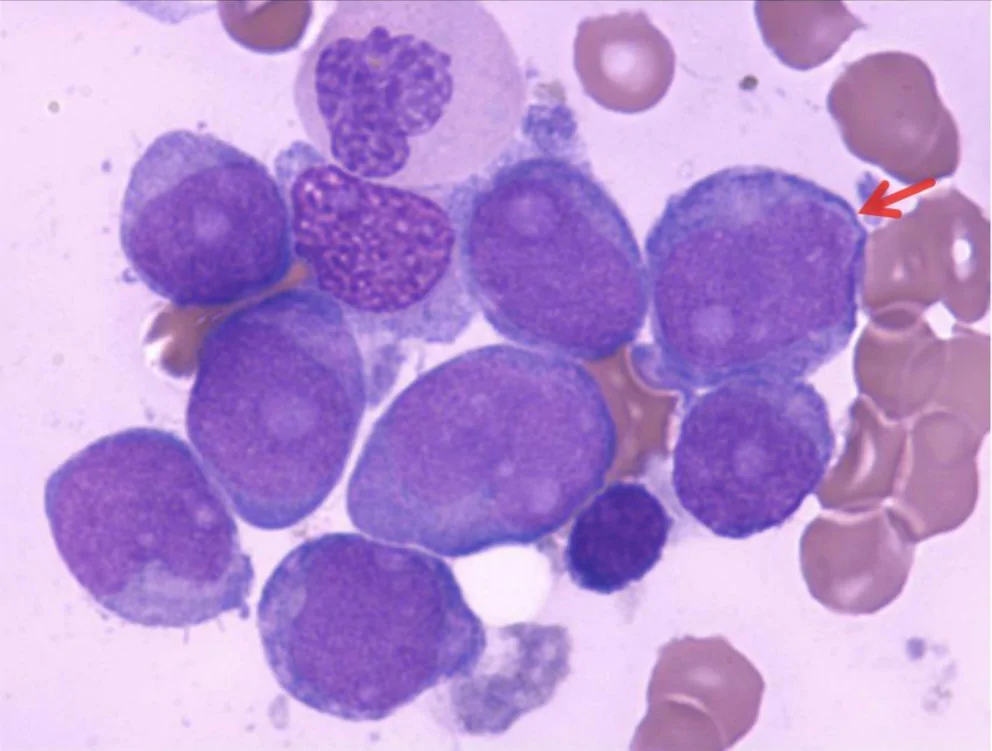
Bone marrow smear (Wright–Giemsa, ×1000). Arrows indicate typical blasts with large cell bodies, grayish‐blue cytoplasm, irregular nuclei, and prominent nucleoli.

3 Flow cytometric immunophenotyping (Figures 2 and 3): The gating strategy is shown in Figure 2. Based on the CD45/SSC dot plot, cells were divided into five subsets: the CD45dim/SSC‐low region (red gate) was defined as blasts, accounting for 83.36% of nucleated cells; the CD45^+^/SSC‐hi region (orange gate) as granulocytes; the CD45^+^/SSC‐int region (green gate) as monocytes; the CD45^+^/SSC‐low region (purple gate) as lymphocytes; and the CD45^−^/SSC‐low region (blue gate) mainly contained mature red blood cells, platelets, and debris. Immunophenotypic analysis of blasts in the CD45dim/SSC‐low region (Figure 3) showed: CD7^−^, CD117^+^, CD13^+^, CD33^+^, CD99^+^, CD34^+^, CD10^−^, CD19^−^, HLA‐DR^+^, cMPO^+^, nTDT^+^, cCD79a^−^, cCD3^−^, CD56^−^, CD38^+^, CD64^−^, CD11c (dim), CD123^+^, CD9^−^, CD4^−^, CD14^−^, CD16^−^, CD65 (dim), CD2^−^, CD11b^−^, CD36^−^.

**FIGURE 2:**
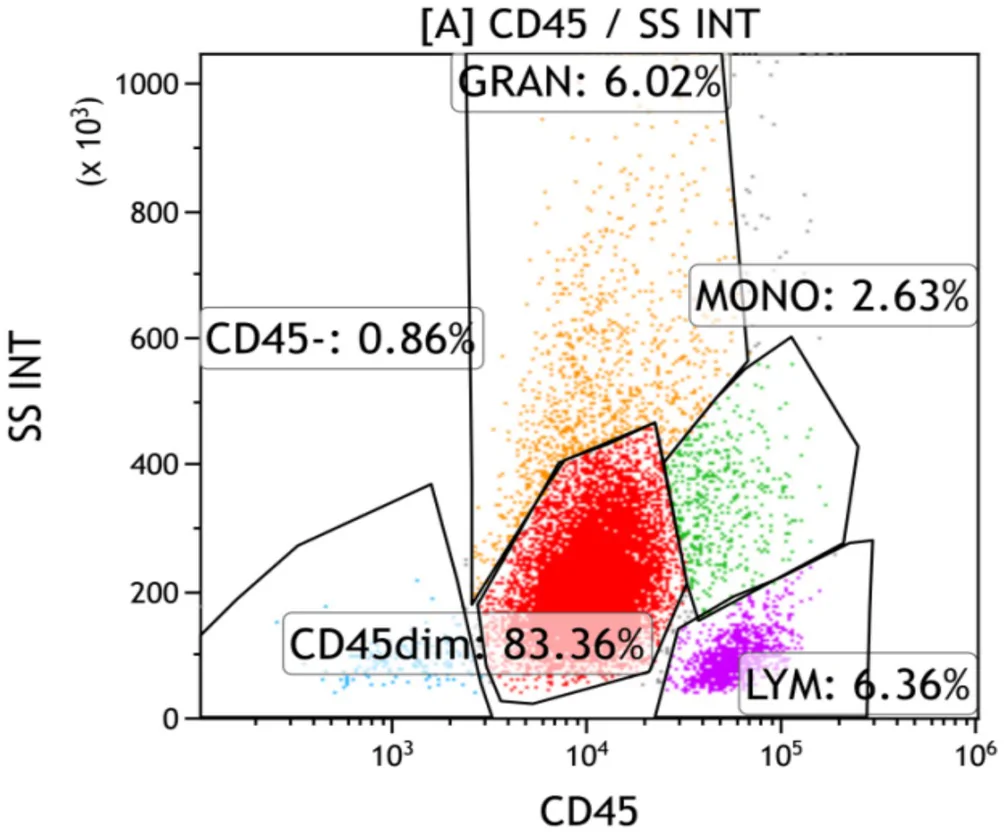
GRAN (orange): Granulocytes; MONO (green): Monocytes; LYM (purple): Lymphocytes; CD45dim (red): Blast region, blasts accounted for 83.36% of nucleated cells; CD45^−^ (blue): Region mainly consisting of mature red blood cells, platelets, and debris, with no blasts.

**FIGURE 3:**
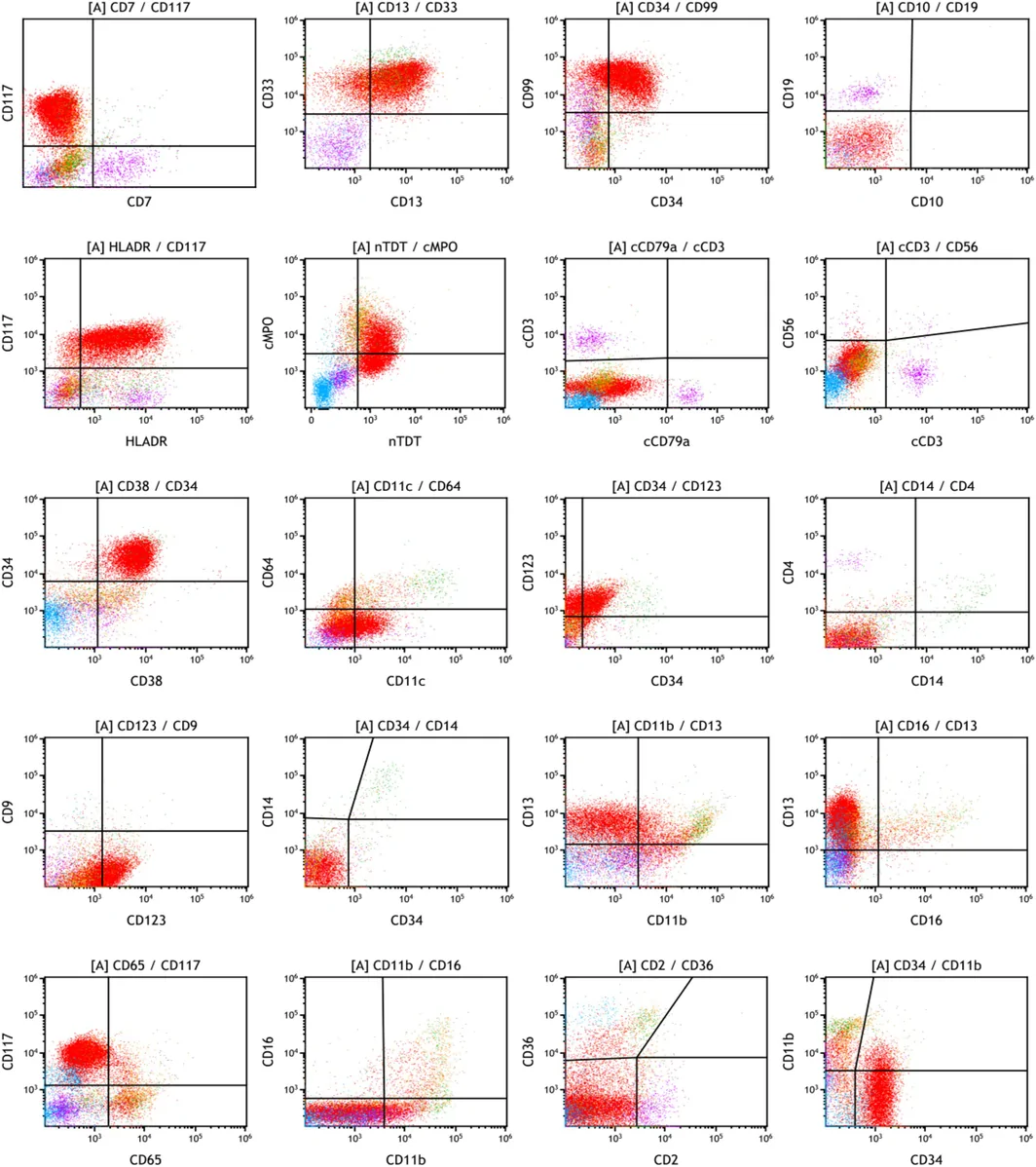
Immunophenotype of blasts (Red) in the CD45dim/SSC‐low region: CD7^−^, CD117^+^, CD13^+^, CD33^+^, CD99^+^, CD34^+^, CD10^−^, CD19^−^, HLA‐DR^+^, cMPO^+^, nTDT^+^, cCD79a^−^, cCD3^−^, CD56^−^, CD38^+^, CD64^−^, CD11c (dim), CD123^+^, CD9^−^, CD4^−^, CD14^−^, CD16^−^, CD65 (dim), CD2^−^, CD11b^−^, CD36^−^.

4 Karyotype analysis (Figure 4): Conventional G‐banding karyotype analysis of 20 metaphase spreads revealed 46,XX,t(6;9)(p23;q34).

**FIGURE 4:**
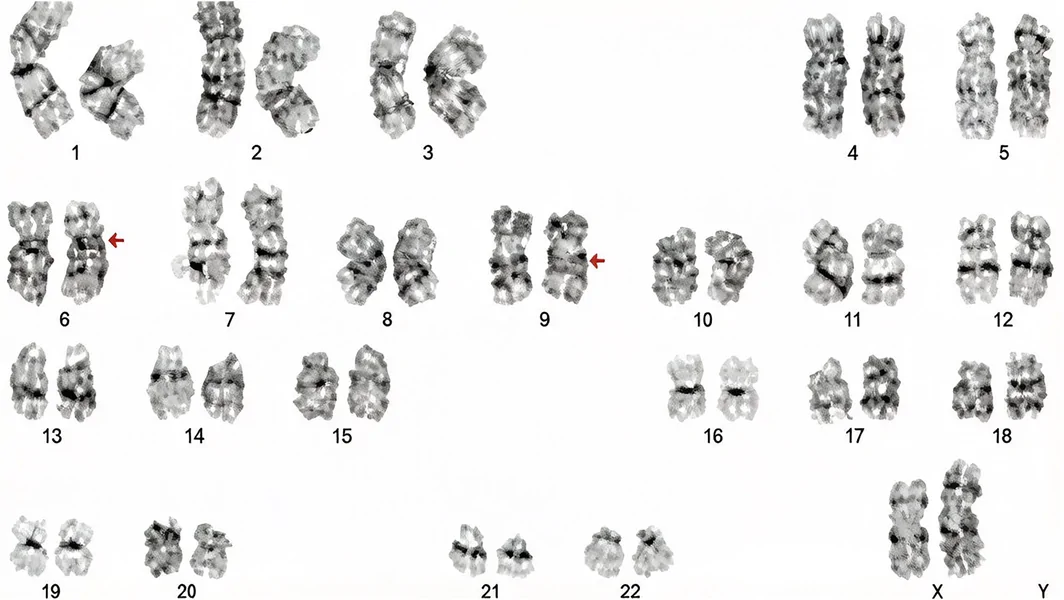
Bone marrow G‐banding karyotype: 46,XX,t(6;9)(p23;q34). Arrows indicate the translocation breakpoints on chromosomes 6 and 9.

5 Molecular testing: In our center, for patients with suspected leukemia, a “56‐leukemia‐related fusion gene” qualitative screening panel (Shanghai Yuanqi Bio) is routinely performed. This test is based on multiplex real‐time reverse transcription PCR (multiplex RT‐PCR coupled with real‐time fluorescence detection using TaqMan probes) and interprets fusion gene positivity/negativity by amplification curves and cycle threshold (CT) values. The screening suggested DEK::CAN fusion gene positivity. For quantitative verification of the fusion gene transcript level, real‐time quantitative PCR (qPCR) with ABL as an internal reference was performed, yielding a DEK::CAN/ABL ratio of 188.21%. The same screening panel also includes quantitative detection of the WT1 gene; the result showed a WT1/ABL ratio of 52.90% (normal reference < 2.5%) [11], indicating WT1 mRNA overexpression. In addition, targeted next‐generation sequencing (NGS, Shanghai Yuanqi Bio, Illumina platform) was used to examine hotspot regions of 22 AML/MDS‐related genes. Sequencing data were analyzed by a standard bioinformatics pipeline (including quality control, alignment, and variant calling). Variants with an allele frequency ≥ 5% were considered positive. A non‐synonymous mutation in exon 6 of the U2AF1 gene (c.467G>A, p.Arg156His) was detected with a variant frequency of 43.30%. No common mutations such as FLT3, RAS, IDH2, DNMT3A, or TET2 were found [3].

### 2.3 Treatment Course and Outcome

Treatment rationale: Based on karyotype t(6;9)(p23;q34) and DEK::CAN fusion gene positivity, the patient was classified as having high‐risk AML. According to the NCCN and ELN guidelines, intensive chemotherapy induction followed by allogeneic hematopoietic stem cell transplantation (allo‐HSCT) is recommended for high‐risk AML [12]. Therefore, we selected the IA regimen (idarubicin + cytarabine) as induction chemotherapy.

The patient received two cycles of IA induction chemotherapy from August 26, 2021. After the first cycle, bone marrow blasts decreased to 25.5% (no remission); after the second cycle (November 22, 2021), bone marrow blasts were 2%, and peripheral white blood cell and platelet counts normalized, achieving complete remission (CR). During this period, she developed a pulmonary fungal infection and *Klebsiella pneumoniae* sepsis, which were controlled with anti‐infective therapy.

Because the patient had acute monocytic leukemia (AML‐M5), which carries a higher risk of central nervous system relapse, lumbar puncture with intrathecal injection was performed on November 23, 2021, to prevent CNS leukemia [13]. She then received three cycles of intermediate‐dose cytarabine consolidation (6 g/cycle). After the first consolidation cycle (December 28, 2021), she developed transient CR with incomplete hematologic recovery (CRi); the final assessment (February 7, 2022) showed re‐established CR. During consolidation, she developed Gram‐negative bacillus sepsis, which was controlled with anti‐infective therapy.

After the third consolidation cycle, the patient developed severe myelosuppression with extreme thrombocytopenia (platelet count 19 × 10^9^/L). In the early morning of February 22, 2022, she suddenly lost consciousness, and bloody vomitus was observed at the bedside; active resuscitation failed, and she died. Clinical discussions considered that the probable cause of death was acute respiratory and circulatory failure due to fatal bleeding (gastrointestinal, intracranial, or pulmonary hemorrhage). Since no autopsy was performed, the exact cause of death could not be confirmed. The bone marrow remained in morphologic remission before death. Owing to recurrent infections and cumulative chemotherapy toxicity, the patient never undergone allo‐HSCT. Detailed treatment timelines and bone marrow/peripheral blood data are presented in Table 1.

**TABLE 1:** Treatment timeline, bone marrow response, and peripheral blood status.

Date	Event/treatment	Bone marrow blasts (%)	Peripheral blood (WBC [×10^9^/L]/Hb [g/L]/PLT [×10^9^/L])	Response assessment	Main complications/notes
2021/8/19	Admission (initial diagnosis)	81%	0.68/35/23	Untreated	Pancytopenia, MDS‐AML diagnosed
2021/8/26	Cycle 1 IA induction	/	0.31/80/21	Not evaluated (BM not examined)	Idarubicin + cytarabine
2021/9/18	After Cycle 1	25.5%	1.08/85/16	Not remitted	Blasts decreased; febrile neutropenia, pulmonary fungal infection
2021/10/12	Cycle 2 IA induction	/	/	Not evaluated	Sepsis ( *Klebsiella pneumoniae* )
2021/11/22	LP + intrathecal chemotherapy	2%	4.30/95/543	Complete remission (CR)	CNS prophylaxis
2021/11/24	Cycle 1 intermediate‐dose cytarabine consolidation	/	/	/	Voriconazole prophylaxis
2021/12/3	Post‐consolidation evaluation	/	0.38/78/40	Not evaluated	Leukopenia, G‐CSF, rhTPO, platelet transfusion
2021/12/28	LP + intrathecal injection	0.5%	2.02/84/87	CR with incomplete hematologic recovery (CRi)	CNS prophylaxis
2021/12/30	Cycle 2 intermediate‐dose cytarabine consolidation	/	/	/	Continued voriconazole
2022/1/9	Post‐consolidation infection	/	0.11/63/37	Not evaluated	Gram‐negative sepsis, controlled with cefoperazone‐sulbactam
2022/2/8	Final BM assessment	0.5%	2.43/104/226	CR	CNS prophylaxis
2022/2/9	Cycle 3 consolidation (cytarabine)	/	/	/	Continued voriconazole
2022/2/18	Post‐chemotherapy myelosuppression	/	0.14/83/19	Severe pancytopenia	G‐CSF, rhTPO, platelet transfusion
2022/2/22	Death	/	/	/	Sudden loss of consciousness, resuscitation failed; clinically presumed fatal bleeding (no autopsy)

*Note:* CR (complete remission) was defined as bone marrow blasts < 5%, with peripheral blood neutrophils > 1.0 × 10^9^/L and platelets > 100 × 10^9^/L; CRi (CR with incomplete hematologic recovery) was defined as bone marrow blasts < 5% but neutrophils < 1.0 × 10^9^/L and/or platelets < 100 × 10^9^/L. This patient achieved CR twice (on November 22, 2021, and February 8, 2022) and had a transient CRi on December 28, 2021.

## 3 Discussion

DEK::CAN is a rare but well‐recognized adverse prognostic marker in AML [14]. Literature review showed that Fang et al. reported a multicenter study of 107 t(6;9)/DEK‐NUP214‐positive myeloid neoplasms (33 MDS, 74 AML), comparing clinicopathologic features and prognosis between MDS and AML; they found no significant difference in overall survival between MDS and AML, but allogeneic HSCT significantly improved survival [3]. Visconte et al. performed detailed molecular profiling of nine patients with isolated t(6;9) AML/MDS, finding that 83% carried at least one somatic mutation (including FLT3‐ITD, NRAS, WT1, etc.), with a median overall survival of only 20 months [10].

As delineated in Table 2, this case represents a rare instance of DEK::CAN‐positive AML with a clearly documented history of longstanding MDS. Notably, it is the first reported case of MDS‐AML characterized by the concomitant presence of DEK::CAN fusion and WT1 mRNA overexpression. Literature data indicate that the 5‐year OS with chemotherapy alone is only 0%–5%, whereas patients who received allo‐HSCT achieved a 5‐year OS of 40%–45% [3] suggesting that transplantation is a key intervention to improve prognosis in this patient population.

**TABLE 2:** Key characteristics of the present case compared with representative cases from the literature.

Feature	Fang et al. [3]	Visconte et al. [10]	Present case
Sample size	107 (33 MDS, 74 AML)	9 (3 MDS, 6 AML)	1
Median age (years)	43 (MDS 51, AML 38)	41	57
Multilineage dysplasia	70% (MDS 90%, AML 61%)	56% (5/9)	Yes
Basophilia (> 2%)	Rare (only 7%)	None	No
Prior MDS history	33 cases (31%)	3 cases (33%)	Yes (at least 5 years)
WT1 status	Mutation (AML 13%, MDS 25%)	Mutation (1 frameshift)	Overexpression (RNA 52.90%), no mutation
FLT3‐ITD frequency	77% (MDS 53%, AML 85%)	33% (2/6)	Not detected
Transplant rate	64% (69/107)	67% (6/9)	0% (not transplanted)
Post‐transplant median OS	62 months	Not calculable (small sample)	Not applicable
Non‐transplant median OS	17 months	/	~12 months
Post‐transplant 5‐year OS	~40%–50%	Not evaluable	Not applicable

*Note:* ① In the study by Fang et al., the transplanted group had a median overall survival (OS) of 62 months, and the 5‐year OS estimated from the Kaplan–Meier curve was approximately 40%–50%; the non‐transplanted group had a median OS of only 17 months, with a 5‐year OS close to 0% [3]. ② The Visconte et al. cohort was too small to provide reliable 5‐year survival data [10]. ③ In the present case, transplantation was not performed because of recurrent infections and chemotherapy toxicity; non‐transplant survival was approximately 12 months, similar to the non‐transplant results in the literature. ④ Multilineage dysplasia: In the Fang cohort, 90% of MDS patients and 61% of AML patients had dysplasia in ≥ 2 lineages; in the Visconte cohort, 56% had multilineage dysplasia; the present case showed trilineage dysplasia (granulocytic, erythroid, and megakaryocytic). ⑤ Basophilia: Fang et al. reported only 7% of patients with peripheral blood/bone marrow basophils > 2%; the Visconte cohort had no basophilia; the present case also showed no basophilia.

It should be noted that the fungal infection and sepsis during treatment were common complications of chemotherapy‐induced myelosuppression and do not indicate treatment failure. In fact, the patient achieved CR after the second induction cycle and maintained bone marrow remission during subsequent consolidation. Moreover, the progression from MDS to AML‐M5 was the natural course of the disease and was unrelated to the subsequent IA regimen. Therefore, the treatment choice in this case was consistent with current guidelines for high‐risk AML, and the infectious complications should not be interpreted as treatment failure.

An important observation in this case is the dissociation between bone marrow response and clinical tolerability. The patient achieved CR after induction, had only a transient CRi during consolidation, and regained CR at the final evaluation. However, recurrent severe infections (fungal infection, bacterial sepsis) and cumulative chemotherapy toxicity consistently prevented her from undergoing allo‐HSCT, and she eventually died of fatal bleeding. This pattern suggests that for DEK::CAN‐positive MDS‐AML, although chemotherapy can effectively clear bone marrow blasts, treatment‐related complications may impede timely transplantation and, in this case, were clinically suspected to contribute to the fatal outcomeTherefore, once any form of remission (CR or CRi) is achieved, allo‐HSCT should be performed as soon as possible under controlled infection and adequate organ function, without waiting for complete hematologic recovery [4, 15]. For future similar patients, this case indicates that intensive chemotherapy is not inherently ineffective, but supportive care (prophylactic antifungal therapy, early anti‐infection measures, platelet transfusions) should be strengthened to reduce complication risks, and transplantation should be promptly bridged after first remission; for those unable to undergo transplantation because of toxicity, alternative strategies such as mTOR inhibitors or WT1‐targeted therapy should be explored.

In recent years, the functional intersection of DEK and WT1, two of the most frequently overexpressed oncogenes in AML, at the level of RNA alternative splicing has attracted attention. Studies have shown that both DEK and WT1 can physically interact with the spliceosome core component U2AF1/U2AF2 heterodimer, thereby affecting the alternative splicing of numerous genes involved in hematopoietic differentiation and chemotherapy resistance [16]. Beyond RNA splicing, recent studies have further revealed that DEK::CAN itself is an XPO1‐dependent transcriptional activator that directly binds to and activates the promoters of FOXC1 and HOXA/B gene clusters, establishing a unique transcriptome signature in t(6;9) AML [17]. Meanwhile, WT1 has been shown to upregulate HOXA3 in an isoform‐specific manner, leading to G1 cell cycle arrest and driving broad chemotherapy resistance [18]. Moreover, DEK::CAN directly activates signaling pathways driven by SRC family kinases (SFK) and mTOR and participates in STAT5 activation [19], while WT1 promotes the self‐renewal of leukemia‐initiating cells by upregulating the anti‐apoptotic proteins BCL2 and BCL2L2 [20, 21]. The present case concurrently harbors the DEK::CAN fusion gene, WT1 mRNA overexpression, and a U2AF1 non‐synonymous mutation (c.467G>A, p.Arg156His, 43.30%). Given that both DEK and WT1 interact with U2AF1 protein, the U2AF1 mutation may enhance or synergize with DEK/WT1 in the aberrant regulation of alternative splicing, adding to HOX reprogramming, apoptosis evasion, and chemotherapy resistance, together shaping a highly aggressive and chemotherapy‐tolerant disease phenotype. Of note, the patient's initial WT1/ABL ratio was as high as 52.90%; although dynamic monitoring was not performed, the literature indicates that persistent WT1 elevation predicts relapse [22, 23, 24] Dynamic monitoring of WT1 levels before treatment, after chemotherapy, and around the time of transplantation is recommended to assess efficacy, predict relapse, and guide preemptive intervention. Furthermore, preclinical studies have shown that mTOR inhibitors (e.g., everolimus) can suppress DEK::CAN‐positive cell growth [5, 25], and WT1‐targeted immunotherapy is under development [8, 26], offering potential low‐toxicity treatment directions for patients unsuitable for transplantation.

This study has several limitations: the DEK::CAN status at initial MDS diagnosis was unknown, precluding determination of whether the fusion gene was an early driver or acquired at transformation; dynamic monitoring of WT1 and MRD was not performed; the cause of death was clinically inferred without autopsy; therefore, it is not possible to definitively distinguish whether death resulted from disease progression or treatment‐related complications; and the patient did not undergo transplantation, so the actual benefit of allo‐HSCT in this case could not be evaluated. In addition, although DEK::CAN fusion gene and WT1 mRNA overexpression may be associated with disease progression, this single‐case report cannot establish causality. Further functional experiments and larger‐scale studies are needed to verify whether they directly drive MDS‐to‐AML transformation.

## 4 Conclusion

DEK::CAN positivity and WT1 mRNA overexpression together define a rare, highly aggressive subtype of MDS‐AML. This case demonstrates that although intensive chemotherapy can induce CR, recurrent infections and cumulative chemotherapy toxicity often prevent patients from undergoing transplantation, leading to death from fatal bleeding rather than leukemia progression. Literature data confirm that allo‐HSCT significantly improves survival (post‐transplant 5‐year OS approximately 40%–50%), whereas the 5‐year OS of non‐transplanted patients is extremely low (close to 0%) [3]. Thus, this case provides important implications for future clinical practice: in the management of DEK::CAN‐positive MDS‐AML, the efficacy of chemotherapy should not be obscured by complications, and strengthening supportive care with timely bridging to transplantation is key to improving prognosis. Specific recommendations are as follows: ① All newly diagnosed MDS patients should routinely undergo karyotype analysis and DEK::CAN fusion gene screening [3]; ② once any form of remission (CR or CRi) is achieved, allo‐HSCT should be promptly bridged under controlled infection and adequate organ function, without waiting for complete hematologic recovery [4]; ③ for patients ineligible for transplantation, low‐toxicity alternative strategies such as WT1‐targeted immunotherapy or mTOR inhibitors should be explored [5, 8, 25, 26]. Future multicenter studies and functional experiments are needed to elucidate the synergistic pathogenic mechanism of DEK::CAN and WT1 and to improve the long‐term prognosis of these patients. Therefore, developing highly selective targeted agents that reduce damage to normal immune cells and lower infection risk is a critical direction for optimizing treatment strategies and improving outcomes in AML.

## Author Contributions

**Wanxiu Mao:** data curation, writing – original draft, investigation, methodology, formal analysis. **Tengmin Zhou:** data curation, resources. **Xi Xu:** data curation, resources, validation. **Sheng Luo:** data curation, resources, validation. **Songfu Jiang:** data curation, resources, methodology, supervision. **Zhijie Yu:** project administration, writing – review and editing, funding acquisition, supervision.

## Funding

This work was supported by Basic public welfare research projects of Wenzhou Science and Technology Bureau (Grant No. Y20240969).

## Ethics Statement

This study was approved by the Ethics Committee of The First Affiliated Hospital of Wenzhou Medical University (Approval No. KY2026‐R0005).

## Consent

Written informed consent was obtained from the patient's family for publication of this case report and accompanying images, in compliance with the Declaration of Helsinki.

## Conflicts of Interest

The authors declare no conflicts of interest.

## Data Availability

The data that support the findings of this study are available on request from the corresponding author. The data are not publicly available due to privacy or ethical restrictions.
